# The miR-33 gene is identified in a marine teleost: a potential role in regulation of LC-PUFA biosynthesis in *Siganus canaliculatus*

**DOI:** 10.1038/srep32909

**Published:** 2016-09-19

**Authors:** Qinghao Zhang, Cuihong You, Shuqi Wang, Yewei Dong, Óscar Monroig, Douglas R. Tocher, Yuanyou Li

**Affiliations:** 1 Marine Biology Institute & Guangdong Provincial Key Laboratory of Marine Biotechnology, Shantou University, Shantou, Guangdong, China; 2 Institute of Aquaculture, Faculty of Natural Sciences, University of Stirling, Stirling, Scotland, UK

## Abstract

As the first marine teleost demonstrated to have the ability to biosynthesize long-chain polyunsaturated fatty acids (LC-PUFA) from C_18_ PUFA precursors, rabbitfish *Siganus canaliculatus* provides a good model for studying the regulatory mechanisms of LC-PUFA biosynthesis in teleosts. Here the potential roles of miR-33 in such regulation were investigated. The miR-33 gene was identified within intron 16 of the gene encoding sterol regulatory element-binding protein 1 (Srebp1), an activator of LC-PUFA biosynthesis. Expression of miR-33 in rabbitfish tissues correlated with that of *srebp1*, while its expression in liver was highly responsive to ambient salinities and PUFA components, factors affecting LC-PUFA biosynthesis. Srebp1 activation promoted the expression of Δ4 and Δ6 Δ5 fatty acyl desaturases (Fad), key enzymes for LC-PUFA biosynthesis, accompanied by elevated miR-33 abundance in rabbitfish hepatocytes. miR-33 overexpression induced the expression of the two *fad*, but suppressed that of insulin-induced gene 1 (*insig1*), which encodes a repressor blocking Srebp proteolytic activation and has targeting sites of miR-33. These results indicated that miR-33, cooperating with Srebp1, may be involved in regulation of LC-PUFA biosynthesis by facilitating *fad* expression, probably through targeting *insig1*. To our knowledge, this is the first report of the participation of miR-33 in LC-PUFA biosynthesis in vertebrates.

Long-chain (≥C_20_) polyunsaturated fatty acids (LC-PUFA) are highly bioactive forms of PUFA and play a number of crucial roles in physiological and biochemical processes in humans and other animals. More specifically, n-3 LC-PUFA such as eicosapentaenoic (EPA; 20:5n-3) and docosahexaenoic (DHA; 22:6n-3) acids are regarded as promoters of human health through reducing cardiovascular risk, regulating the inflammatory response and promoting neurodevelopment^1^. In vertebrates, LC-PUFA can be biosynthesized from C_18_ PUFA. For example, α-linolenic acid (LNA; 18:3n-3) can be Δ6 desaturated to produce 18:4n-3, which can then be elongated to 20:4n-3 followed by Δ5 desaturation to produce EPA^2^. Biosynthesis of DHA from EPA in vertebrates can occur through two different pathways depending upon species. One is the so-called “Sprecher pathway” that requires two successive elongation steps from EPA to produce 24:5n-3, and a second Δ6 desaturation step to 24:6n-6 that is then chain-shortened to DHA by partial β-oxidation in peroxisomes^3^. The other is the “Δ4 desaturation pathway” where EPA can undergo a single elongation to 22:5n-3, which is then directly Δ4 desaturated to produce DHA^4^. While the ‘Sprecher pathway’ is regarded as the most widespread DHA biosynthesis pathway among vertebrates, the “Δ4 desaturation pathway” was first demonstrated in the rabbitfish *Siganus canaliculatus* and thereafter in other fish species^5^,^6^,^7^. However, humans are inefficient at LC-PUFA biosynthesis and uptake EPA and DHA mainly through consuming fish^8^,^9^. Thus considerable attention has been given to the LC-PUFA biosynthetic pathways and regulation in teleosts, which aims to increase the nutritional benefits of fish to human health whilst tries to replace fish oils (FO), rich in LC-PUFA but limited in resources and high in cost, by sustainable vegetable oils (VO), rich in C_18_ PUFA precursors but devoid of n-3 LC-PUFA, in the feed of cultured fish species of commercial importance^10^,^11^,^12^.

Fatty acyl desaturases (Fad) are key enzymes for LC-PUFA biosynthesis and catalyze the desaturation steps described above^12^. All the teleost *fad* genes cloned to date are homologous to mammalian *fads2*, but their substrate specificities differ among species and monofunctional and bifunctional desaturases with Δ4, Δ5 and Δ6 activities have been described^13^. Thus, the functional expression of *fad* underlies the molecular wiring of the LC-PUFA biosynthetic pathway, which increases interest in the regulation of *fad* expression in teleosts, especially in marine species that are generally regarded as having limited LC-PUFA biosynthetic ability from C_18_ PUFA precursors compared to freshwater and salmonid fish^10^,^12^.

Sterol regulatory element-binding proteins (Srebp) are major transcription factors coordinating lipid synthesis in liver. Srebp are translated as inactive protein precursors that require proteolytic cleavage in Golgi to gain transactivation potency^14^. However, this activation process can be blocked by insulin-induced gene 1 (Insig1), an endoplasmic reticulum (ER) integral membrane protein that retains Srebp precursors in ER membranes. The Srebp family consists of Srebp1 (1a and 1c) and Srebp2 proteins that are encoded by two unique genes^14^. Unlike Srebp2 that is involved in cholesterol metabolism, Srebp1 is a pivotal activator for fatty acid synthesis. Considering LC-PUFA biosynthesis, Srebp1c activates the expression of Δ5 and Δ6 desaturase genes (encoded by *fads1* and *fads2*, respectively) in mouse liver. Similarly, studies in Atlantic salmon (*Salmo salar*) showed that *srebp1* can be activated effectively by TO901317, a liver X receptor (Lxr) agonist, dependent upon the Lxr-Srebp1 pathway, and further promoted the expression of Δ5 and Δ6 *fad in vitro*^15^,^16^.

MicroRNAs (miRNA or miR) are short, non-coding RNA molecules with about 22 nucleotides that regulate gene expression at a post-transcriptional level^17^. Our recent study in rabbitfish demonstrated that miR-17 was involved in regulation of LC-PUFA biosynthesis by repressing liver expression of Δ4 *fad*^18^, and highlighted the importance of miRNAs in regulation of LC-PUFA biosynthesis in vertebrates. Recently, miR-33 has been found within the intron of *srebp* genes and functions in cooperation with its host in mammals^19^,^20^,^21^,^22^,^23^,^24^. For example, miR-33 inhibits cholesterol efflux by down-regulating the expression of ATP binding cassette A1 (Abca1), which facilitates cholesterol synthesis induced by Srebp2^19^,^20^,^21^. However, other than one study in rainbow trout describing the effects of miR-33 in the insulin pathway^25^, little is known about the function of miR-33 in teleosts and, to our knowledge, there are no reports of its involvement in LC-PUFA biosynthesis in any vertebrate.

As a commercially important species, rabbitfish *S. canaliculatus* was the first marine teleost in which all gene activities of desaturation and elongation required to bioconvert C_18_ PUFA precursors into C_20–22_ LC-PUFA were identified and characterized^4^,^26^,^27^. Thus, rabbitfish provides a good model to investigate the regulatory mechanisms of LC-PUFA biosynthesis in teleosts, particularly in terms of promoting *fad* expression. Consequently, the aim of the present study was to investigate the role of miR-33 in regulation of LC-PUFA biosynthesis in teleosts. First, the gene of miR-33 was cloned from an intron of the rabbitfish *srebp1* gene using genome walking technology, which prompted us to investigate its possible role in LC-PUFA biosynthesis. To address this issue, we first determined the expression patterns of miR-33 and *srebp1* in rabbitfish tissues followed by their responses to environmental salinities *in vivo*, and to PUFA treatment *in vitro*, both of which were factors demonstrated to influence LC-PUFA biosynthesis in this species^26^,^28^. Subsequently, the potential role of miR-33 in regulation of LC-PUFA biosynthesis was investigated in rabbitfish primary hepatocytes challenged with TO901317, and further verified by the effect of miR-33 overexpression on *srebp1*, Δ4 *fad*, Δ6 Δ5 *fad*, as well as *insig1* mRNA levels in primary hepatocytes. These data provide us with novel insights into the mechanisms of LC-PUFA biosynthesis regulation in vertebrates, which may contribute to the optimization and/or enhancement of the LC-PUFA pathway in teleosts.

## Results

### Rabbitfish miR-33 is located within intron 16 of *srebp1* gene

**Figure 1:**
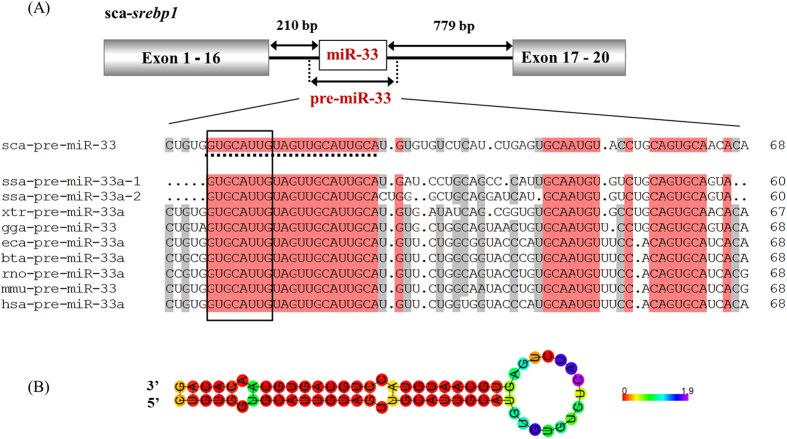
Rabbitfish miR-33 is located within *srebp1* intron and conserved in evolution. (**A**) Genomic location of miR-33 is flanked by 210 bp from exon 16, and 779 bp from exon 17 in the *srebp1* gene. The putative sequence of pre-miR-33 was aligned with its orthologs in human (*Homo sapiens*, hsa), mouse (*Mus musculus*, mmu), rat (*Rattus norvegicus*, rno), horse (*Equus caballus*, eca), cattle (*Bos taurus*, bta), chicken (*Gallus gallus*, gga), frog (*Xenopus tropicalis*, xtr), and salmon (*Salmo salar*, ssa) using ClustalX2, and identical residues are shaded black while similar residues are in grey. Mature miR-33 sequence was dot underlined and the seed sequence (UGCAUUG) is framed. (**B**) Predicted secondary structure of rabbitfish pre-miR-33 with lowest dG value = −32.20 kcal/mol. Different colors denote positional entropy.

The miR-33 gene was cloned from intron 16 of *srebp1* (NCBI accession: KU670831) in rabbitfish (Fig. 1 and Supplementary Table S1) and included mature miR-33 encoding capability (Supplementary Fig. S1). The rabbitfish pre-miR-33 was 69 nucleotides (nt) with the typical stable stem-loop secondary structure (dG = −32.20 kcal/mol) necessary for mature miRNA processing (Fig. 1). The rabbitfish mature miR-33 was 21 nt confirmed by small RNA deep sequencing and showed high identity to mammal miR-33a (Fig. 1 and Supplementary Table S2). At the 5′ end of rabbitfish miR-33, a 7 nt “seed sequence” (UGCAUUG) was identified, which is pivotal for target recognition of miRNA (Fig. 1).

**Figure 2:**
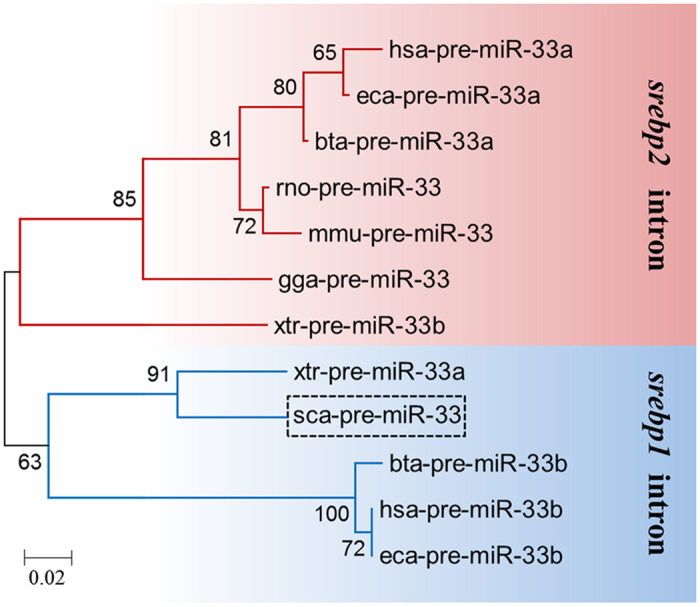
Phylogenetic analysis of pre-miR-33 in combination with their gene locations. A phylogenetic tree was constructed to compare the putative rabbitfish pre-miR-33 nucleotide sequences with their orthologs (a/b isoforms) in different species. Moreover, their locations within the introns of *srebp1*/*2* genes were denoted. The horizontal branch length is proportional to the number of base substitutions per site and indicates evolutionary distances. The numbers represent the frequencies (%) with which the tree topology presented was replicated after 1000 iterations.

Results of phylogenetic analysis showed that rabbitfish pre-miR-33 clustered together with mammal and avian pre-miR-33b orthologs and amphibian pre-miR-33a, all of which are located within introns of the *srebp1* gene, and separate from pre-miR-33a clusters and amphibian pre-miR-33b, which are all present in introns of the *srebp2* gene (Fig. 2).

### miR-33 is co-transcribed with *srebp1* mRNA in rabbitfish tissues

**Figure 3:**
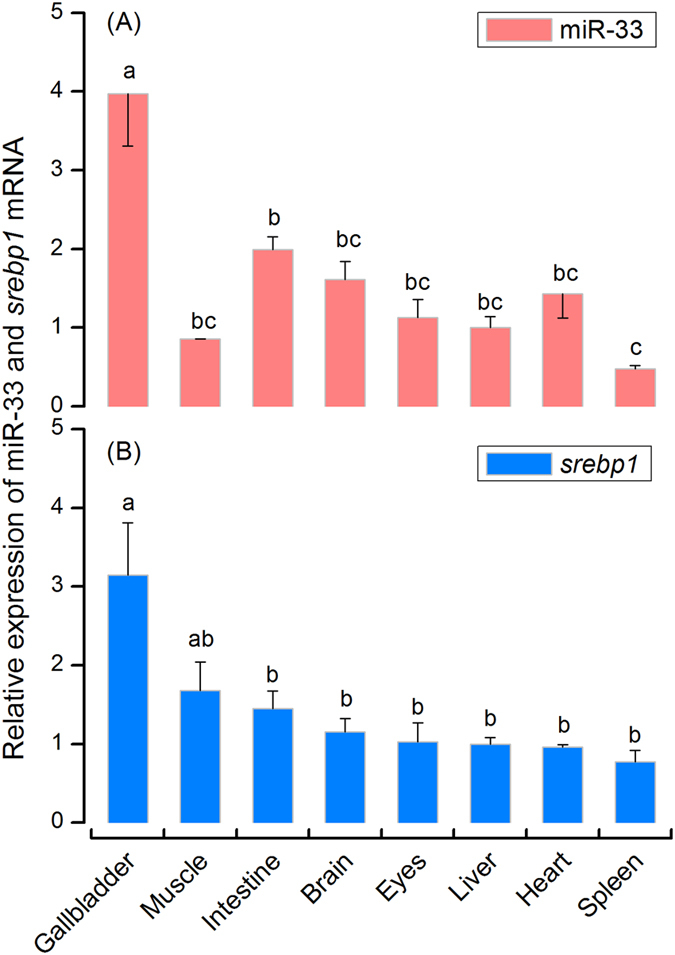
Tissue distribution of miR-33 (**A**) and *srebp1* mRNA (**B**) in rabbitfish. Values are means ± SEM (n = 3) as fold change from the liver, bars without sharing a common superscript indicate significant difference among the detected tissues (*P* < 0.05; ANOVA, Tukey’s test).

To test whether miR-33 can be co-expressed with its host *srebp1* in rabbitfish, the abundance of miR-33 and *srebp1* mRNA were determined in selected tissues (Fig. 3). Results showed that miR-33 was widely expressed in the examined tissues with highest expression level in gallbladder, lowest in spleen, and intermediate levels in intestine, brain, eyes, liver, heart and muscle. Furthermore, miR-33 displayed parallel expression with *srebp1* in all tissues except muscle.

### Hepatic miR-33 showed coordinated expression pattern with *srebp1* mRNA both *in vivo* and *in vitro*

**Figure 4:**
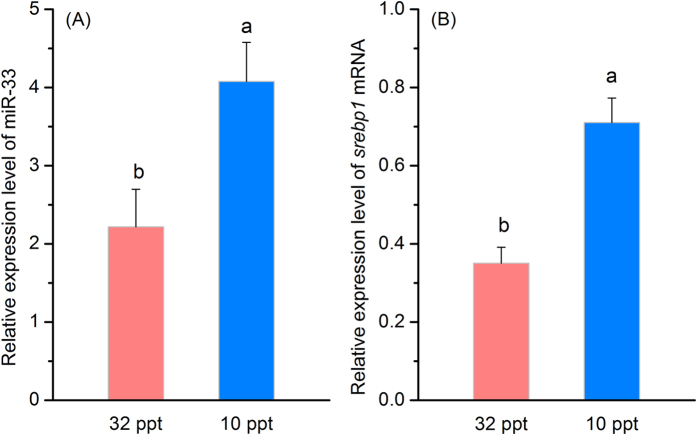
Expression of miR-33 and *srebp1* in livers of rabbitfish reared at 32 ppt and 10 ppt. The expression levels of miR-33 (**A**) and *srebp1* (**B**) were determined by qPCR. Data are means ± SEM (n = 6) and different superscripts indicate significant differences (*P* < 0.05; ANOVA, Tukey’s test).

**Figure 5:**
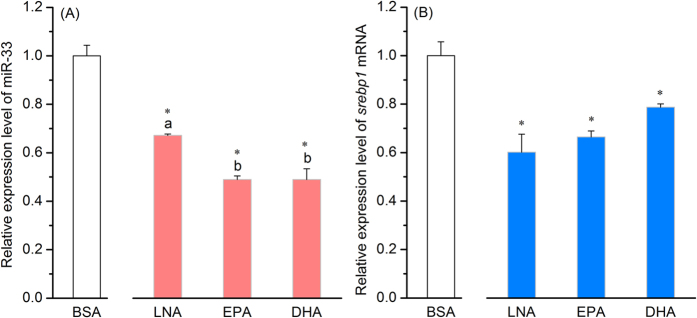
Expression of miR-33 and *srebp1* mRNA in rabbitfish hepatocytes incubated with PUFA. The isolated rabbitfish primary hepatocytes were incubated with 18:3n-3 (LNA), 20:5n-3 (EPA) or 22:6n-3 (DHA) and bovine serum albumin (BSA) as control. The relative RNA levels of miR-33 (**A**) and *srebp1* mRNA (**B**) were assessed by qPCR. Data were presented as the fold change from control in means ± SEM (n = 3). Different superscripts above columns denote significant differences among PUFA treatment groups while asterisks represent significant differences between each fatty acid treatment and BSA control (*P* < 0.05; ANOVA, Tukey’s test).

The previous studies have well-demonstrated that the expression of *fad* and LC-PUFA biosynthetic ability were higher in liver of rabbitfish reared at 10 ppt salinity or fed C_18_ PUFA-rich diets (like LNA) than that of fish reared at 32 ppt or fed C_20–22_ LC-PUFA rich diets (like EPA and DHA)^26^,^28^. In the present study, miR-33 and *srebp1* displayed similar expression patterns *in vivo* with higher transcript abundance in liver of rabbitfish reared at 10 ppt than that at 32 ppt (*P* < 0.05) (Fig. 4). *In vitro*, the expression of both miR-33 and *srebp1* were significantly (*P* < 0.05) decreased in rabbitfish hepatocytes incubated with any of the PUFA tested (LNA, EPA and DHA) compared to BSA controls, confirming the validity of the assay. Interestingly, the transcript abundances of miR-33 were higher in hepatocytes when incubated with LNA compared to EPA and DHA (*P* < 0.05) (Fig. 5).

### Srebp1 activation promoted the *fad* expression, accompanied by increasing abundance of miR-33

**Figure 6:**
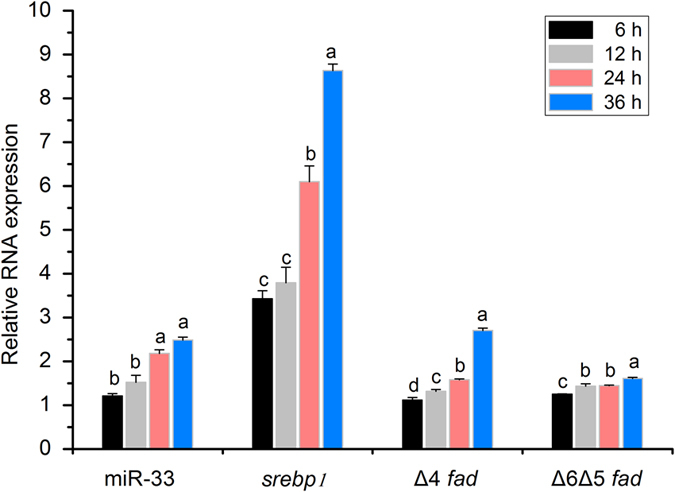
Srebp1 activation induced the expression of miR-33, Δ4 *fad* and Δ6Δ5 *fad* in rabbitfish primary hepatocytes. The gene expression was determined by qPCR in rabbitfish primary hepatocytes treated with TO901317 from 6 h to 36 h. Data are presented as fold change from the DMSO control at detected time points in means ± SEM (n = 3). Asterisks represent significant differences (*P* < 0.05; ANOVA, Tukey’s test).

According to the coordinated expression pattern of both miR-33 and *srebp1*, we investigated the potential role of miR-33 in regulation of LC-PUFA biosynthesis by treating rabbitfish primary hepatocytes with TO901317, an Lxr agonist that activates the expression of *srebp1* through the Lxr-Srebp1 pathway. Results showed that the expression level of *srebp1*, miR-33, and Δ4 *fad* increased gradually from 6 h to 36 h after TO901317 supplementation, whereas transcription of Δ6 Δ5 *fad* increased at 36 h (*P* < 0.05) (Fig. 6).

### miR-33 induced *fad* expression and showed targeting effects on *insig1* expression

**Figure 7:**
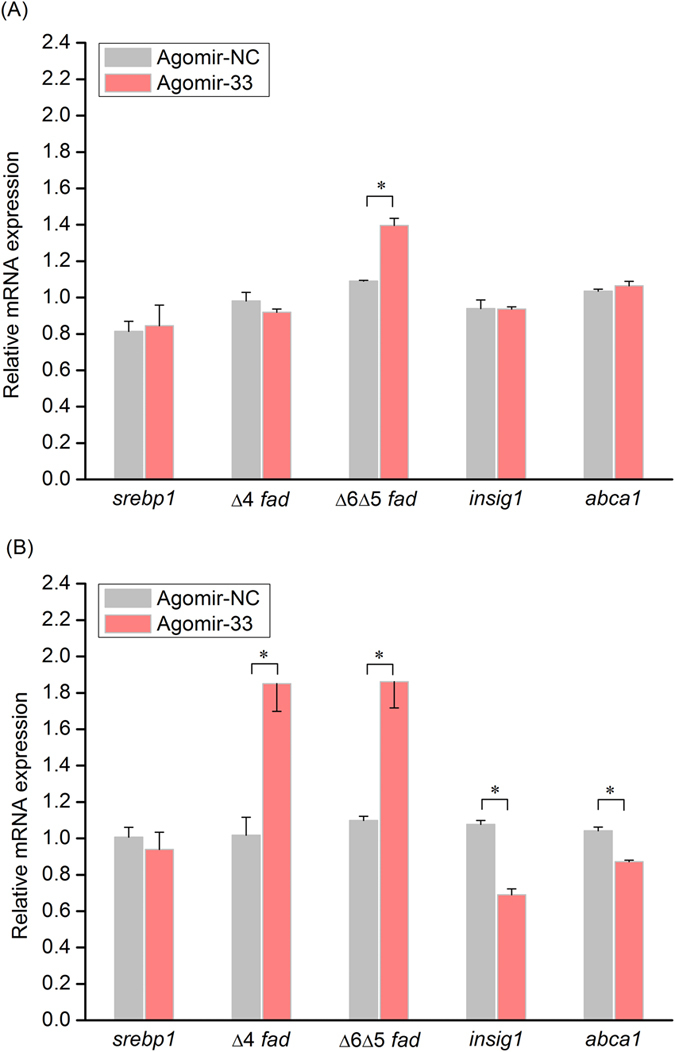
Effects of miR-33 overexpression on the mRNA level of *srebp1*, Δ4 *fad*, Δ6 Δ5 *fad, insig1* and *abca1* in rabbitfish primary hepatocytes. The gene expression was determined by qPCR in rabbitfish primary hepatocytes transfected with AgomiR-33 or Agomir-NC for 12 h (**A**) and 24 h (**B**). Data are presented as fold change from the mock control at detected time points in means ± SEM (n = 3). Asterisks represent significant differences (*P* < 0.05; ANOVA, Tukey’s test).

**Figure 8:**
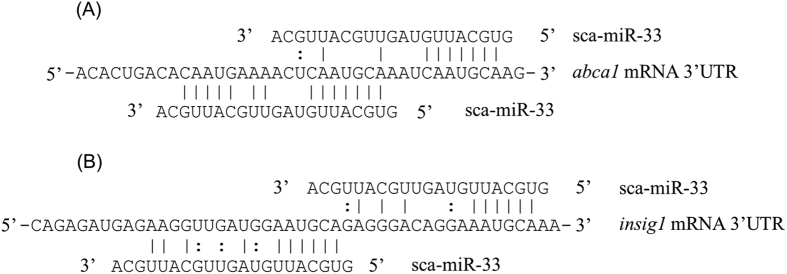
Scheme of miR-33 base pairing the 3′UTR of the rabbitfish *abca1* mRNA (**A**) and *insig1* mRNA (**B**).

The role of miR-33 in regulation of expression of genes involved in LC-PUFA biosynthesis was further determined by overexpression (Agomir-33) experiments. Results showed that the overexpression of miR-33 first induced the transcription of Δ6 Δ5 *fad* at 12 h (Fig. 7) and this was further enhanced at 24 h, while the transcription of Δ4 *fad* was induced at 24 h (*P* < 0.05) (Fig. 7). In contrast, the expression of *abca1* (positive control) was suppressed at 24 h (*P* < 0.05), which confirmed the effectiveness of Agomir-33. The level of *insig1* mRNA was also decreased at 24 h after Agomir-33 transfection (*P* < 0.05). In accordance with the down-regulation of *insig1* and *abca1* expression, adjacent target sites for miR-33 in the 3′ UTR of *insig1* mRNA were predicted (Fig. 8). In addition, the transcription of *srebp1* was not significantly influenced by Agomir-33 transfection at either time point (Fig. 7).

## Discussion

In mammals, miR-33 exists as two distinct isoforms, namely miR-33a and b, which differ from each other in two bases outside the seed region of the mature versions^19^. More specifically, human miR-33b is embedded in intron 17 of the *srebp1* gene while miR-33a is present in intron 16 of the *srebp2* gene. In mouse, a unique miR-33 gene has been found within intron 15 of the *srebp2* gene and displayed high conservation with human miR-33a. However, our knowledge of miR-33 was very limited in teleosts. On one hand, this can be attributed to the sporadic appearance of miR-33 gene in teleost genomes. For example, miR-33 is present in Atlantic salmon with three homologs, but absent in zebrafish (*Danio rerio*) and carp (*Cyprinus carpio*)^29^,^30^,^31^. On the other hand, the relatively limited availability of genomic databases from teleosts, especially for commercially important species, hampers *in silico* miR-33 searches. In the present study, we cloned and identified the miR-33 gene from intron 16 of *srebp1* in rabbitfish using genome walking technology, which, to our knowledge, is a novel application in miRNA studies.

In animals, the primary transcripts of miRNA genes (pri-miRNA) are processed sequentially into precursor miRNA (pre-miRNA, ~70 nt) and mature miRNA (~22 nt)^32^. While mature miRNA emerge as functional regulators, here we propose that pre-miRNA sequences can reflect the genomic location of miRNA genes. In the present study, rabbitfish pre-miR-33 was clustered with pre-miR-33b orthologs from mammals, all located within *srebp1* introns, although the sequence of rabbitfish miR-33 was conserved with mammal miR-33a orthologs that are present within introns of *srebp2*. Thus, the phylogenetic analysis of pre-miR-33 might provide molecular clues for miR-33 gene localization in other fish species in the absence of extensive genomic sequence information. However, further studies are required to confirm this hypothesis for other miRNA categories. In addition, only one miR-33 mature form has been identified in rabbitfish in the present study. A similar situation has been observed in Atlantic salmon where distinct pre-miR-33a and b were processed into a unique mature miR-33 form with an identical sequence to mammal miR-33a^31^. Given the evolutionary position of teleosts, these results indicated that miR-33a and b share a common molecular ancestry in evolution and their complete divergence may occur after the division of aquatic-terrestrial lineages. To some extent, this can be supported by the evidence from *Drosophila melanogaster* where miR-33, homologous to mammalian miR-33a, is embedded in intron of the *srebp* gene that is unique through whole genomes^33^, while the concurrent emergence of mature miR-33a and b initiates at amphibians, according to data from miRBase^34^.

Intronic miRNA are always coordinately transcribed with their host genes from a common transcript^35^,^36^,^37^, and miR-33 was reported to co-express with *srebp* in human tissues^19^,^20^. Consistently, the rabbitfish miR-33 was expressed in parallel with *srebp1* in all tissues examined except muscle. Similar results were observed in human skeletal muscle and chicken thigh and breast muscle^19^,^38^. This may be attributed to miRNA editing after transcription. For example, selective editing reduced the abundance of miR-142 and miR-151 by inhibiting the processing of pri-miR-142 and pre-miR-151, which further affected their tissue-specific expression^39^,^40^. However, the exact mechanism for the low abundance of miR-33 in muscle merits further investigation.

Fad are the rate-limiting enzymes in the pathway of LC-PUFA biosynthesis^12^. Our previous studies showed that the expression levels of Δ4 and Δ6 Δ5 *fad* genes, as well as the overall LC-PUFA biosynthetic ability, were higher in liver of rabbitfish reared at 10 ppt or fed a VO diet rich in C_18_ PUFA like LNA, compared to those reared at 32 ppt salinity or fed a FO diet containing high levels of EPA and DHA^26^,^28^. Consistent with increased expression of Δ4 and Δ6Δ5 *fad* genes at low salinities (10 ppt), the expression of miR-33 responded similarly and displayed higher abundance in liver of fish maintained at 10 ppt compared to 32 ppt. In addition, higher expression levels of miR-33 were observed in rabbitfish hepatocytes incubated with LNA compared to EPA and DHA. These results indicated strongly that miR-33 is involved in regulation of LC-PUFA biosynthesis in teleosts.

In mammals, miR-33 functions in cooperation with its host Srebp in regulation of lipid metabolism^19^,^20^,^21^,^23^,^41^. In rabbitfish, miR-33 was present within intron 16 of *srebp1*, a master transcription factor activating *fad* transcription in liver^15^,^16^,^42^,^43^, and thus we focused on the potential effects of miR-33 on *fad* expression, which is completely unstudied to date. In rabbitfish hepatocytes treated with TO901317, Δ4 *fad* was more sensitive than Δ6 Δ5 *fad* to Srebp1 activation, accompanied by elevated miR-33 abundance. Moreover, miR-33 overexpression also promoted Δ4 *fad* expression and further enhancement of Δ6 Δ5 *fad* expression in rabbitfish hepatocytes at 24 h after Agomir-33 transfection. Therefore, miR-33 may play a promoting role in LC-PUFA biosynthesis by facilitating the expression of *fad*, particularly Δ4 *fad*, in cooperation with its host Srebp1.

Typically, miRNA inhibit the functional expression of target genes at a post-transcriptional level through partial complementarity to the 3′ untranslated regions (UTR), which results in translation repression and/or mRNA decay^17^. However, emerging studies demonstrated that miRNAs can also activate gene expression directly^44^,^45^,^46^. The liver-specific miR-122 increased the transcription of hepatitis C virus by complementing to its 5′ UTR^46^, while miR-369-3p binds to the 3′ UTR of tumor necrosis factor-α and activates its translation in quiescent G0 cells^45^. However, there is no potential binding site for miR-33 predicted in either 5′ or 3′ UTR of the two *fad* mRNAs and so we hypothesized that miR-33 functions indirectly by repressing negative regulators for *fad* expression. Studies in mammals showed that Insig1 blocked the proteolytic cleavage of Srebp proteins by retaining Srebp precursors in the ER membrane and consequently decreased lipogenesis^47^,^48^,^49^. Interestingly, we found two adjacent putative binding sites for miR-33 in the 3′UTR of the rabbitfish *insig1* mRNA and, moreover, demonstrated that the mRNA level of *insig1* was decreased by miR-33 overexpression. Therefore, it is tempting to speculate that miR-33 decreases the expression of *insig1*, thus facilitating the proteolytic activation of Srebp1 protein that further activates *fad* expression in rabbitfish. This explanation would also account for the apparent lack of response in *srebp1* mRNA abundance after miR-33 overexpression. However, the exact mechanism of interaction between miR-33 and *insig1* mRNA, and their physiological significance still requires further investigation.

In summary, the present study indicated that, as an intronic miRNA in cooperation with Srebp1, rabbitfish miR-33 may be involved in regulation of LC-PUFA biosynthesis by facilitating the expression of *fad*, probably through targeting *insig1* mRNA post-transcriptionally. To our knowledge, this is the first report of the participation of miR-33 in LC-PUFA biosynthesis in vertebrates.

## Materials and Methods

### Ethics statement

In present study, all procedures performed on fish were in accordance with the National Institutes of Health guide for the care and use of Laboratory animals (NIH Publications No. 8023, revised 1978) and approved by the Animal Care and Use Committee of Shantou University (Guangdong, China). All surgery was performed under 0.01% 2-phenoxyethanol (Sigma-Aldrich, St. Louis, MO, USA) anesthesia, and all efforts were made to minimize suffering of fish.

### Animals and tissue sampling

The rabbitfish and samples used in the present study have been described in detail previously^18^. Briefly, 500 rabbitfish juveniles (body mass ~13 g, sex indistinguishable visually) were captured from the coast near Nan Ao Marine Biology Station (NAMBS) of Shantou University, Southern China. After one month of adaption to the aquarium, half of the fish were acclimated from original seawater (32 ppt) to brackish water (10 ppt) for one month, while the remaining individuals were maintained in seawater. After a further two-week acclimation, an eight-week feeding trial was carried out. Throughout the trial, all fish were fed a single formulated feed consisting of 32% crude protein and 8% crude lipid, a blend of fish oil (FO) and vegetable oil (VO). During the pre-trial acclimation period, the liver from one rabbitfish was sampled for later DNA isolation and cloning, and tissue samples from another three individual fish were collected for later determination of miR-33 and *srebp1* tissue-specific expression. At the initiation of the feeding trial, livers of three fish were collected as initial controls and, at the end of the trial, livers of six fish (two individuals per triplicate tank) per salinity treatment were sampled for comparative analysis of gene expression. Tissue samples were immediately frozen in liquid nitrogen and subsequently stored at −80 °C until further use.

### Molecular cloning of miR-33 gene in rabbitfish

Due to the absence of rabbitfish genomic data, the gene of miR-33 was obtained by genome walking technology. Briefly, genomic DNA prepared from rabbitfish liver was used as template (DNeasy^®^ blood & tissue kit, Qiagen, Hilden, Germany). Subsequently, the miR-33 partial gene sequence was amplified by miR-33-part-F/R primers with high fidelity *Pfu* polymerase (Tiangen Biotech, Beijing, China), followed by its up-stream sequence being cloned using genome walking technology (Takara, Dalian, China) with two gene-specific primers (miR-33-ups-sp1 and miR-33-ups-sp2) and arbitrary primers provided by the kit. By alignment with rabbitfish *srebp1* cDNA sequence, a 306 bp fragment was overlapped with the up-stream of the miR-33 gene. Accordingly, a pair of primers (srebp1-intron-F/R), flanking the miR-33 gene location, was designed to determine the intron sequence including miR-33 using high fidelity *Pfu* polymerase. Finally, the specific location of miR-33 was determined by alignment with the complete sequence of the *srebp1* gene that was cloned by PrimeSTAR^®^ HS DNA Polymerase (Takara, Dalian, China). All primer sequences are shown in Supplementary Table S3.

### Sequence and phylogenetic analysis of miR-33

To further verify the identity of miRNA and understand its evolutionary context, sequence and phylogenetic analysis were conducted. The putative precursor miR-33 (pre-miR-33) sequence of rabbitfish was compared with its corresponding orthologs from human (accession: MI0000091), mouse (MI0000707), rat (MI0000874), horse (MI0009807), cattle (MI0009807), chicken (MI0001170), frog (MI0004922) and salmon (ssa-miR-33a-1, MI0026690; ssa-miR-33a-2, MI0026691) in miRBase (http://www.mirbase.org/) using ClustalX2 sequence alignment, and the secondary structure was retrieved by RNAfold online (http://rna.tbi.univie.ac.at/). Phylogenetic trees were constructed on the basis of nucleotide sequence alignments between rabbitfish pre-miR-33 and their orthologs using Neighbour Joining method with MEGA 5^50^.

### Rabbitfish primary hepatocyte isolation and incubation with PUFA or TO901317

In order to study the gene expression of miR-33 and *srebp1* in rabbitfish liver *in vitro*, primary hepatocytes were isolated and cultured as recently described^18^. Briefly, rabbitfish were fasted for 24 h prior to anesthesia with 0.01% 2-phenoxyethanol and sacrifice. Excised liver tissue was dispersed by 0.1% collagenase (Gibco, Life Technologies, USA)/0.25% hyaluronidase (Sigma-Aldrich, St. Louis, USA) and subsequently primary hepatocytes with viability ≥95% were plated on 6-well plates coated with 0.1% gelatin at a density of 2 × 10^6^ cells per well in DMEM/F12 (Gibco, Life Technologies, USA) containing 20% fetal bovine serum (FBS), 100 U/ml penicillin and 100 μg/ml streptomycin, followed by incubation for 24 h at 24 °C/4% CO_2_. PUFA/BSA complexes including LNA, EPA and DHA (Cayman Chemical Co., Ann Arbor, USA) were prepared as 5 mM stock solution according to Ou *et al*.^51^. TO901317 (Sigma-Aldrich, St. Louis, MO, USA), an Lxr agonist activating Lxr-Srebp1 pathway, was prepared as a stock solution (10.39 mM): 5 mg TO901317 powder was dissolved in DMSO in the manufacturer’s bottle and stored at −20 °C. Primary hepatocytes were incubated for 1 h in FBS-free DMEM/F12 and then exposed to fresh DMEM/F12 medium containing LNA, EPA or DHA at 100 μM for 6 h or 2 μM TO901317 for 6 h, 12 h, 24 h and 36 h, respectively. In addition, 0.1% BSA and DMSO was used as control for PUFA and TO901317 treatments, respectively. Within each incubation experiment, treatments were performed in triplicate wells. For cell harvest, incubation media were removed and then cells were lyzed in wells using lysis buffer provided by the RNAprep pure cell/bacteria kit (Tiangen Biotech, Beijing, China), followed by RNA extraction according to the manufacturer’s instructions.

### Transfection of rabbitfish primary hepatocytes with miR-33 mimics

To further investigate the potential role of miR-33 in regulation of gene expression in LC-PUFA biosynthesis, endogenous miR-33 was up-regulated by transfection with miR-33 mimics (FAM-labeled). First, rabbitfish primary hepatocytes were purified by Percoll density gradient (40% Percoll, density 1.05–1.06 g/ml) at 3220 g for 3 min^52^. Subsequently, cells were washed twice with ice-cold HBSS and then suspended in FBS-free DMEM/F-12 at a density of 1 × 10^6^ cells/ml. For transfection, Lipofectamine^®^ RNAiMAX Reagent (Invitrogen, Carlsbad, CA, USA) was used with 10 nM miR-Up^TM^ Agomir-33 (miR-33 mimics), or scrambled negative controls (Agomir-NC) (Genepharma, Shanghai, China) in six-well plates, respectively. In addition, cells incubated with only transfection reagent served as the mock control for normalizing gene expression. Finally, cells were washed with ice-cold PBS, lysed and harvested for later RNA extraction and qPCR detection at 12 h and 24 h after Agomir transfection. *abca1*, a well-documented and conserved target for miR-33, was used as a positive control gene to estimate the efficacy of miR-33 mimics^19^,^20^,^21^,^41^.

### RNA isolation and quantitative real-time PCR (qPCR) determinations

For extraction of total RNA from rabbitfish tissues, Trizol reagent (Invitrogen, Carlsbad, CA, USA) was utilized, whereas for primary hepatocytes, total RNA was isolated and purified by RNAprep pure cell/bacteria kit (Tiangen Biotech, Beijing, China). The concentration and quality of total RNA were confirmed by spectrophotometry (NanoDrop 2000, Thermo Scientific, USA) followed by cDNA synthesis from 1 μg of total RNA from rabbitfish tissues, 500 ng from chemically incubatory hepatocytes, and 100 ng from post-transfected rabbitfish primary hepatocytes, respectively (miScript II RT Kit, Qiagen, Hilden, Germany). The expression of miR-33 was determined using the miScript SYBR Green PCR Kit (Qiagen, Hilden, Germany) with miR-33 specific primer (qPCR-miR-33) and universal primer provided in the kit. For qPCR measurement of *srebp1* (NCBI accession: JF502069.1), Δ4 *fad* (GU594278.1), Δ6 Δ5 *fad* (EF424276.2), *insig1* (KU598855) and *abca1* (KU587554) mRNA expression level, LightCycler^®^ 480 SYBR Green I Master (Roche, Germany) was used with rabbitfish gene-specific primers (Supplementary Table S3). The relative RNA level of each sample were normalized to *18s* rRNA (AB276993) and calculated by the comparative threshold cycle method^53^. For the analysis of relative gene expression in the livers of rabbitfish from 10 ppt and 32 ppt salinity, the Ct value of initial liver samples from three fish was used as control. All reactions were run in LightCycler^®^ 480 thermocycler (Roche, Germany) using qPCR programs according to manufacturer’s specifications.

### Statistical analysis

All the data were checked for homogeneity of variance using Levene’s test. Differences among groups were analyzed by one-way analysis of variance (ANOVA) followed by Tukey’s multiple comparison test at a significance level of *P* < 0.05 using OriginPro 7.5 software (OriginLab Corporation, Northampton, MA, USA). All the data were presented as means ± SEM (n = 6 for the analysis of liver gene expression in rabbitfish reared at 10 ppt or 32 ppt; n = 3 for the analysis of tissue-specific distribution and gene expression in cell assays).

## Additional Information

**How to cite this article**: Zhang, Q. *et al*. The miR-33 gene is identified in a marine teleost: a potential role in regulation of LC-PUFA biosynthesis in *Siganus canaliculatus. Sci. Rep.* **6**, 32909; doi: 10.1038/srep32909 (2016).

## Supplementary Material

Supplementary Information
