# Supplementary material for: The miR-33 gene is identified in a marine teleost: a potential role in regulation of LC-PUFA biosynthesis in Siganus canaliculatus
Source: Sci Rep. 2016 Sep 19;6:32909. doi: 10.1038/srep32909 (PMC5027541; doi:10.1038/srep32909)
Supplement: Supplementary Information [file srep32909-s1.pdf]

Title

The miR-33 gene is identified in a marine teleost: a potential role in regulation of LC-PUFA biosynthesis in *Siganus canaliculatus*

Authors

Qinghao Zhang [a†], Shuqi Wang [a†], Cuihong You [a], Yewei Dong [a], Óscar Monroig [b], Douglas R. Tocher [b], Yuanyou Li [a*]

Supplementary Table S1. The mRNA to genomic sequence alignment of rabbitfish *srebp1*.

Exon	Genomic coordinates (bp)	mRNA coordinates (bp)	Length (bp)
Exon 1	1-306	1-306	306
Exon 2	2670-3230	307-867	561
Exon 3	4543-4691	868-1016	149
Exon 4	4820-4954	1017-1151	135
Exon 5	5099-5317	1152-1370	219
Exon 6	5411-5525	1371-1485	115
Exon 7	5785-5942	1486-1643	158
Exon 8	6045-6261	1644-1860	217
Exon 9	6367-6551	1861-2045	185
Exon 10	6663-6924	2046-2307	262
Exon 11	7038-7204	2308-2474	167
Exon 12	7359-7527	2475-2643	169
Exon 13	7649-7757	2644-2752	109
Exon 14	7871-7980	2753-2862	110
Exon 15	8775-8901	2863-2989	127
Exon 16	9037-9208	2990-3161	172
Exon 17	10219-10439	3162-3382	221
Exon 18	10926-11031	3383-3488	106
Exon 19	11473-11718	3489-3734	246
Exon 20	12933-13111	3735-3913	179

Supplementary Table S2. The mature sequence of miR-33 and its expression level in the liver of rabbitfish determined by small RNA deep sequencing.

Sequence	Counts	RPM (mean number of reads per million)
GTGCATTGTAGTTGCATTGCA	1560	165

Supplementary Table S3. Primers used for gene clone and qPCR

Aim	Gene/sequence name	Primers/oligonucleotides	Nucleotide sequence
Partial gene clone	Gene of miR-33	miR-33-part-F	5'- GCTGTGGTGCATTGTAGTTG-3'
		miR-33-part-R	5'- GCGGCGTCTGTGTTGC-3'
Genome walking PCR	Gene of miR-33 and upstream	miR-33-ups-sp1	5'- CTGTGTTGCACTGCAGGTACAT-3'
Full length PCR	Intron 16 of *srebp1* gene	srebp1-intron-F	5'- CTGCTCCGAGACAGCCTCAAC-3'
		srebp1-intron-R	5'- GCCACACGTTGGTCCTCATCA-3'
		miR-33-ups-sp2	5'- GCAGGTACATTGCACTCAGATGAGACA-3'
qPCR	miR-33	qPCR-miR-33	5'- CGTGCATTGTAGTTGCATTGC-3'
	srebp1	qPCR-srebp1-F	5'- CGGAGCCAAGACAGAGGAGTG-3'
		qPCR-srebp1-R	5'- GTCTCCCAGCTTCTCCAAGGTAC-3'
	Δ4 *fad*	qPCR-Δ4 fad-F	5'- TGCACCTAACAGGAGTCACGTT-3'
		qPCR-Δ4 fad-R	5'- GCTCAGCACAGGATTGAGTACG-3'
	Δ6Δ5 *fad*	qPCR-Δ6Δ5 fad-F	5'- TCACTGGAACCTGCCCACAT-3'
		qPCR-Δ6Δ5 fad-R	5'- TTCATTCTCAGACAGTGCAAACAG-3'
	insig1	qPCR-insig1-F	5'- AGTTACGCCGCCTGAGAAGA-3'
		qPCR-insig1-R	5'- TGGGCATCACCACTGCTTTC-3'
	abca1	qPCR-abca1-F	5'- CTTGTGGTTGATTGATGTCTGTG-3'
		qPCR-abca1-R	5'- ATCCGCTCGCCTCTTAGTTC-3'
	18s rRNA	qPCR-18s-F	5'- CGCCGAGAAGACGATCAAAC-3'
		qPCR-18s-R	5'- TGATCCTTCCGCAGGTTCAC-3'

(A)

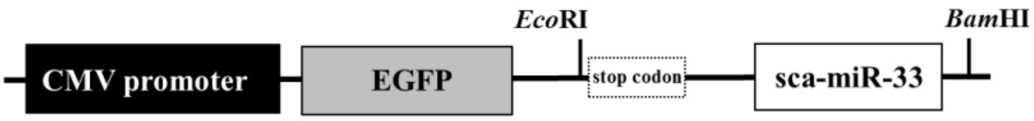

(B)

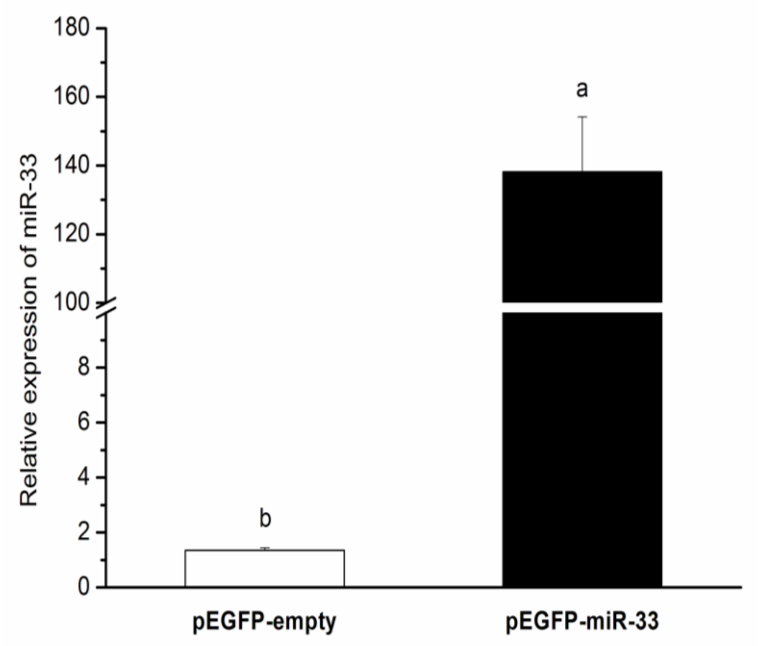

Supplementary Fig. S1. Heterogeneous expression of miR-33 from intron 16 of rabbitfish *srebp1* gene in HEK293T cells. (A) Schematic representation shows the construction of miR-33 expression vector (pEGFP-miR-33). A 369 bp DNA fragment encompassing miR-33 gene from intron 16 of rabbitfish *srebp1* gene was cloned into the pEGFP-C3 plasmid behind an artificial stop codon by using *EcoR*I and *BamH*I enzymes. (B) The HEK 293T cells were transfected with pEGFP-miR-33 or pEGFP-empty (pEGFP-C3 plasmid with no insert), and then the expression level of miR-33 was determined by qPCR. Data are means ± SEM (n=3) and bars with different superscripts are significantly different from each other ($P < 0.05$; ANOVA, Tukey's test).
